# Genipin-Crosslinked Gelatin Hydrogels with Controlled Molecular Weight: A Strategy to Balance Processability and Performance

**DOI:** 10.3390/gels11120980

**Published:** 2025-12-05

**Authors:** Ángela Castro-María, Juan P. Fernández-Blázquez, Jennifer Patterson

**Affiliations:** 1 IMDEA Materials Institute, 28906 Getafe, Madrid, Spain; angela.castro@imdeamaterials.org (Á.C.-M.); juanpedro.fernandez@imdeamaterials.org (J.P.F.-B.); 2 Bioengineering Department, Universidad Carlos III de Madrid, 28911 Leganés, Madrid, Spain

**Keywords:** gelatin, hydrogels, genipin crosslinking, biocompatibility, drug delivery, tissue engineering, rheology

## Abstract

Gelatin-based hydrogels are promising materials for pharmaceutical and biomedical applications due to their biocompatibility, biodegradability, and tunable gel-forming behavior. However, their thermo-sensitivity and limited processability often restrict their practical use in advanced drug delivery or tissue engineering systems. In this study, low-molecular-weight gelatin (LMWG) was obtained from native gelatin through controlled degradation with hydroxylamine, aiming to enhance processability while maintaining functional amino groups for crosslinking. Hydrogels prepared from both native gelatin and LMWG were crosslinked with genipin, a natural and biocompatible compound, and comprehensively characterized in terms of structural, mechanical, and biological properties. LMWG exhibited superior processability, remaining liquid at room temperature, which facilitates the preparation of different formulations and the potential incorporation of bioactive compounds into the crosslinked hydrogels. Compared with gelatin-genipin hydrogels, LMWG-genipin hydrogels showed higher swelling capacity, slightly increased porosity, and improved flexibility without significant loss of mechanical integrity. Rheological analysis confirmed both hydrogels’ viscoelastic properties with differences in their thermo-sensitive behavior. Cytocompatibility assays using L929 fibroblasts demonstrated low toxicity as well as proliferation of cells seeded on the materials. Overall, the combination of molecular weight modulation and crosslinking by genipin provides a simple and effective strategy to develop gelatin-based hydrogels suitable for pharmaceutical formulations, tissue-engineering scaffolds, and controlled-release systems.

## 1. Introduction

Tissue engineering is a multidisciplinary field combining biological, physical, and chemical knowledge to develop constructs based on three principal elements: biomaterials, cells, and growth factors. The main objective is to produce biological substitutes to repair, replace, or regenerate damaged organs or tissues in the human body. For that, it is very important to develop three-dimensional (3D) structures, known as scaffolds, to support the growth of cells in or on them. Bioprinting is emerging as one of the most promising techniques to produce complex 3D structures through layer-by-layer deposition of a bioink, which contains cells and growth factors inside [1]. Bioinks are usually based on natural or synthetic polymers, with one of their main formats being hydrogels [2]. Hydrogels are hydrophilic crosslinked networks that can keep a large amount of water inside [3], and the crosslinked structure prevents their dissolution in aqueous media. Three main types of hydrogels are possible [3,4]: hydrogels with a physically crosslinked structure in which reversible molecular interactions produce the crosslinks, a chemically crosslinked structure with covalent bonds between the polymeric chains, and finally a hybrid structure with both physical and chemical crosslinks.

Hydrogels present numerous properties that make them interesting materials for biomedical and pharmaceutical applications. They often exhibit excellent biocompatibility and are similar to the extracellular matrix (ECM) in terms of morphology, mechanical properties, and water content. In addition, they can favor the attachment and migration of cells [4]. Furthermore, hydrogels possess a micro- to nano-porous structure that allows the introduction of biological substances. Some hydrogels can retain compounds via molecular interactions, whereas others can be covalently functionalized with biomolecules of interest [5]. To prepare stable, chemically crosslinked hydrogels, different synthetic compounds, such as glutaraldehyde, formaldehyde, epoxy compounds, dialdehydes, and isocyanates, have been used as crosslinkers. Nevertheless, these reagents often compromise biocompatibility [6,7,8]. For that reason, other compounds have been investigated, for example, genipin [9,10]. Genipin is a natural crosslinker extracted from *Gardenia jasminoides Ellis* [9]. It is used in the biomedical field because it forms biocompatible and stable products and presents low cytotoxicity [11]. Genipin has previously been used as a crosslinker of collagen, gelatin, polyethylene glycol, and chitosan to make tissue engineering scaffolds [12,13] or to use for drug delivery [14,15].

One of the materials that is commonly used to produce hydrogels due to its potential in the biomedical field is gelatin, which is a partially denatured protein obtained from the acid or alkaline hydrolysis of collagen, resulting in gelatin Type A or B, respectively [16,17,18]. Although the mechanism of crosslinking gelatin with genipin is not proven, this reaction is believed to occur in two steps, as proposed by Butler et al. [9]. The first step consists of a nucleophilic attack that permits the formation of a stable intermediate, and the second step involves the nucleophilic substitution of a free amine group on the lysine side chain of gelatin into the activated ester group of genipin [9,19,20]. On the one hand, genipin-crosslinked gelatin hydrogels are being researched in the field of tissue engineering [21] for ocular [20], bone [12], or cartilage [13] applications. On the other hand, gelatin crosslinked with genipin has attracted significant attention as a stable and non-toxic carrier system for the controlled release of bioactive molecules. Recent studies have demonstrated that such hydrogels can modulate the release kinetics of therapeutic agents, such as anti-inflammatory drugs, by adjusting crosslinking conditions, thereby ensuring sustained and localized delivery while maintaining excellent cytocompatibility [22]. Moreover, gelatin-based microspheres have been used as delivery vehicles for growth factors in tissue engineering applications. For example, they have been shown to effectively promote chondrogenic differentiation in stem cell cultures by providing localized and sustained release of cytokines like transforming growth factor (TGF)-β1 [23]. These findings collectively support the potential of genipin-crosslinked gelatin systems as versatile platforms for controlled drug delivery and pharmaceutical applications. Nevertheless, gelatin has some disadvantages, as gelatin solutions can be very viscous and they require processing temperatures above body temperature, typically between 40 and 50 °C to maintain a liquid state.

To solve this problem, one possibility that is being researched for the first time in this work is the use of low-molecular-weight gelatin (LMWG) that is then crosslinked with genipin to form hydrogels. LMWG is obtained from gelatin Type A or B through a degradation process with hydroxylamine, as initially described by Piluso et al. [24]. The degradation process has been shown to result in protein fragments with molecular weights of 15, 25, 37, and 50 kDa, independent of the time and conditions in which the reaction occurs. The main advantage of LMWG, as shown in previous studies [24,25], is that it is liquid in aqueous solvents at room temperature. In contrast, gelatin in aqueous solvents at room temperature is solid, hindering its manipulation. In other words, LMWG has a lower sol–gel transition temperature than gelatin.

This study prepares hydrogels from gelatin and LMWG by crosslinking them with a fixed concentration of genipin. First, a characterization of the molecular weight and the quantification of the free amino groups present in gelatin and LMWG is carried out. Then, both hydrogels are compared through dynamic mechanical analysis (DMA) and rheology to determine their mechanical properties along with evaluation of their swelling capacity. Finally, cell culture experiments are carried out to determine the possible cytotoxicity of the hydrogels, as well as a morphological study of both the hydrogels and the cells interacting with them.

## 2. Results and Discussion

### 2.1. LMWG Synthesis and Characterization

**Figure 1:**
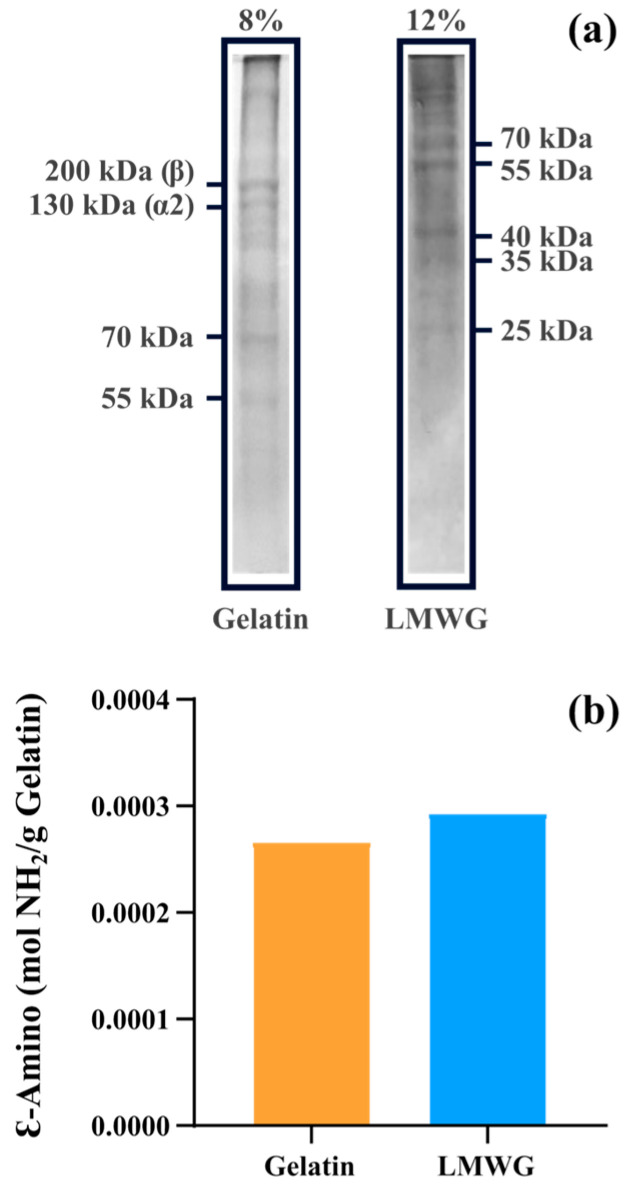
Characterization of gelatin and LMWG. (**a**) SDS-PAGE results with 8% and 12% polyacrylamide gels for gelatin and LMWG, respectively. (**b**) Concentration in mol/g of Ɛ-amino groups in gelatin and LMWG obtained by the TNBS assay.

Gelatin and LMWG were subjected to sodium dodecyl sulfate–polyacrylamide gel electrophoresis (SDS-PAGE) to assess their molecular weight distributions and the effect of the hydroxylamine degradation of gelatin. The results showed differences in the polymer chains of both materials. Gelatin chains of 200 kDa, 180 kDa, 130 kDa, 70 kDa, and 50 kDa were found, whereas in the case of LMWG, the polymer chains were 70 kDa, 55 kDa, 40 kDa, 35 kDa, and 25 kDa. While bands for the β and α chains of gelatin were clearly visible (Figure 1a), the treatment of gelatin with hydroxylamine led to the complete disappearance of the bands for the β, α1, and α2 chains. These bands of high molecular weight that are typically present in gelatin were absent in all samples of LMWG, indicating effective fragmentation of the polymer chains. The molecular weight distribution of the resulting fragments of LMWG was also consistent with the previous report by Piluso et al. [24], where bands at approximately 50, 37, 20 and 15 kDa were detected. This prior study also thoroughly characterized the LMWG by SDS-PAGE, rheology, wide-angle X-ray scattering (WAXS), and gel permeation chromatography (GPC) and showed that, regardless of reaction time, temperature, or hydroxylamine concentration, the reaction proceeds in a rapid and specific manner [24].

Moreover, 2,4,6-trinitrobenzenesulfonic acid (TNBS) analysis was conducted to quantify the amount of free ɛ-amino groups available within the polymer chains of both materials. The amino groups both from the N-terminus and from the side chains of some amino acids are responsible for reacting with genipin during the hydrogel formation [9]. The amino group concentration in the LMWG samples increased relative to that in the gelatin samples (Figure 1b) and can be attributed to the degradation of gelatin. Treatment of gelatin with hydroxylamine causes a specific cleavage of peptide bonds, particularly between the asparagine (Asn) and glycine (Gly) residues, which are widely present in the gelatin structure [24]. As a result, the polymeric chain is broken, leading to the formation of new-NH_2_ end groups. This increase in the number of free amino groups was experimentally confirmed by the TNBS assay, and the values measured are in line with previous reports [24]. This increase in amino groups suggests that degradation leads to increased availability of reactive sites for crosslinking during hydrogel synthesis. Overall, the chemical characterization of both materials showcased the successful production of the LMWG due to a reduction in molecular weight. Additionally, evaluating the number of amino groups provided information about the influence of the degradation on the crosslinking capacity, with an increase in amino groups available for crosslinking in the LMWG.

### 2.2. Hydrogel Synthesis and Morphology

**Figure 2:**
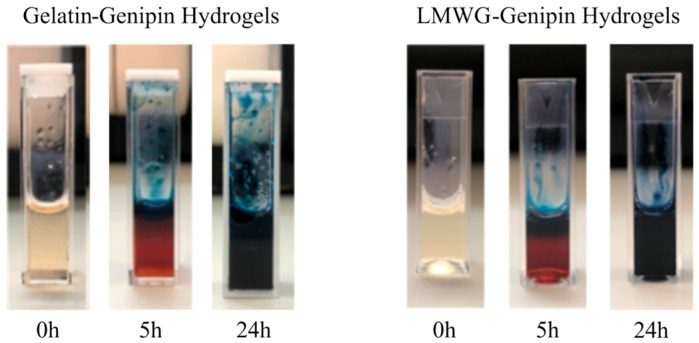
Progressive change of color in the gelatin-genipin and LMWG-genipin hydrogels.

Crosslinking was successfully achieved in both hydrogels within 24 h of initiating the reaction. During this time, a gradual transformation, as indicated in Figure 2, was apparent, with the coloration progressing from a transparent starting solution to a dark blue hydrogel. The evolution in color serves as visual evidence of the successful progression of crosslinking. The color change is due to the oligomerization reaction of products formed by adding genipin to amines. This reaction occurs in addition to the main crosslinking process between genipin and the amino groups present in gelatin. It consists of an oxygen radical-induced polymerization of genipin, accompanied by the dehydrogenation of intermediate compounds generated after genipin ring opening, which is initiated by the nucleophilic attack of a primary amino group. These secondary reactions are responsible for the formation of the characteristic blue pigments observed during the crosslinking process [9,26].

**Figure 3:**
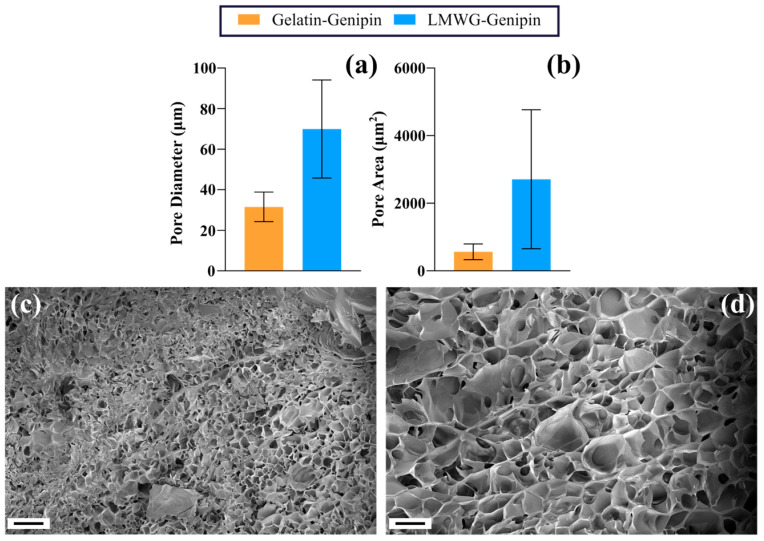
(**a**) Mean maximum pore diameter and (**b**) mean maximum pore area for gelatin-genipin and LMWG-genipin hydrogels. Data were obtained by analyzing 150 pores (50 pores from 3 different SEM images per hydrogel type), first averaging per image, then calculating a global mean (n = 3), including accumulated error. A *t*-test yielded *p*-values of 0.11 for diameter and 0.22 for area. (**c**,**d**) SEM images of gelatin-genipin and LMWG-genipin hydrogels, respectively, highlighting differences in their porous microstructures. Scale bars: 50 µm.

Hydrogels were prepared for observation using scanning electron microscopy (SEM), with the results shown in Figure 3. The images revealed a porous microstructure of both materials after lyophilization, which allows for a comparison between hydrogels of different compositions. Analysis of mean pore diameter and mean pore area showed that the LMWG-genipin hydrogels had larger pores, which averaged 69.96 ± 24.19 μm (mean area 2713.13 ± 2054.59 μm^2^), than those of the gelatin-genipin hydrogels, which were 31.57 ± 7.26 μm (mean area 566.58 ± 233.36 μm^2^). However, these differences between the two hydrogels were not statistically significant. These pore size values fall within the ranges identified as favorable for tissue regeneration. For effective cell growth, hydrogels must contain pores of an appropriate size to allow cell migration through the material [27]. Achieving an optimal pore size is key to promoting cell proliferation and facilitating nutrient and oxygen diffusion throughout the material. Literature reports highlight that pore size strongly influences scaffold functionality, with smaller pores (<100 μm) favoring cell attachment, seeding efficiency, and cell-to-cell communication and larger pores (100–400 μm) enhancing nutrient diffusion, vascularization, and long-term tissue stability [28]. In this study, the LMWG-genipin hydrogels with larger pores (~70 μm) align with structural features reported to facilitate both early cell adhesion and vascularization, suggesting potential applications in bone or other highly vascularized tissues [28,29]. Conversely, the gelatin-genipin hydrogels with smaller pores (~32 μm) provide an architecture more suited to supporting dense cell populations, migration, and intercellular communication, relevant for skin regeneration or lung applications [28,30]. Overall, the porous structures of both lyophilized hydrogels are consistent with the requirements described for functional scaffolds, supporting their potential use in tissue engineering applications. However, it is acknowledged that this method of measuring porosity is not representative of the mesh size of the hydrated hydrogels, as the lyophilization process may alter the pore structure of the hydrogels. During lyophilization, the samples are frozen, and the water contained in the network forms solid crystals, which are then removed by sublimation. This constitutes a methodological limitation, as the porosity observed after freeze-drying does not reflect the original microstructure of the hydrated hydrogel but depends on the size of the ice crystals formed. In addition, the final structure obtained depends on the freezing temperature. Lower temperatures favor nucleation and, therefore, the formation of smaller crystals, resulting in smaller pores [31,32]. Despite this limitation, the results provide valuable insights into the general trends in pore size distribution and their potential effects on cellular interactions.

### 2.3. Swelling Ratio and Degradation Studies and Mechanical Property Measurements

**Figure 4:**
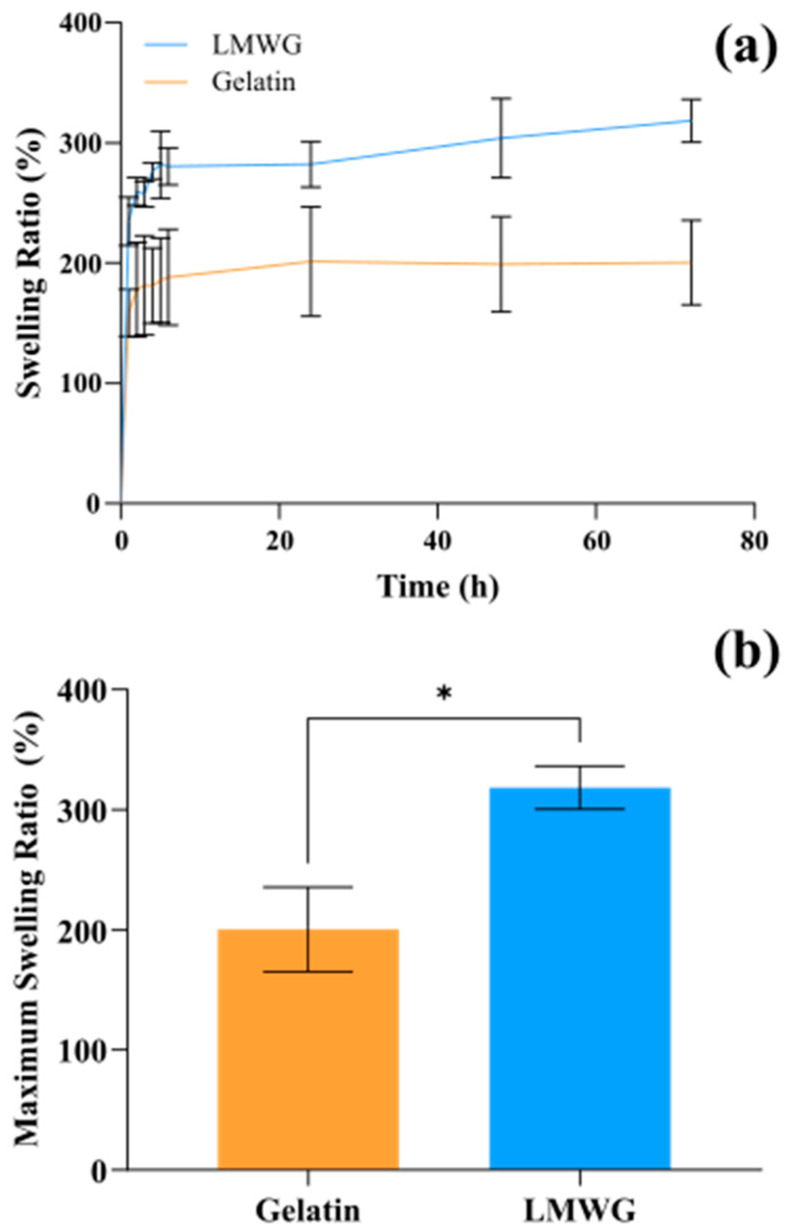
(**a**) Swelling ratio versus time for gelatin-genipin and LMWG-genipin hydrogels, with mean values and error bars showing standard deviation (n = 3). (**b**) Maximum swelling ratio with mean values and error bars showing standard deviation (n = 3). *t*-test * *p*-value ≤ 0.05.

Considering the definition of a hydrogel as a 3D crosslinked polymer network that can absorb and retain large amounts of water [33], the swelling ratio was one of the main properties of these materials to study. As shown in Figure 4, the LMWG-genipin hydrogels had a significantly higher maximum swelling ratio, 318.35 ± 17.78%, than the gelatin-genipin hydrogels, 200.30 ± 35.21% (*p*-value = 0.0066). Considering time-dependent behavior, the swelling of both hydrogels was most pronounced during the first 5 h, as the hydrogels absorbed fluid rapidly. After this initial phase, the swelling ratio values stabilized, reflecting the establishment of an equilibrium between the thermodynamic force exerted by the fluid and the contractile force of the crosslinked polymer chains within the hydrogel structure.

The swelling capacity of hydrogels is primarily determined by the structure of their polymer network, particularly the mesh size (ξ), defined as the average distance between cross-linking points. ξ is a key structural parameter in hydrogels, as it determines the free volume available for penetration and retention of solvent molecules, directly influencing the swelling capacity. Higher ξ promotes solvent absorption, while reduced ξ limits swelling by restricting solvent mobility within the network. In numerous models of solvent diffusion in hydrogels, ξ appears explicitly as a variable since it controls the physical resistance offered by the lattice to the migration of molecules [34]. In the LMWG-genipin hydrogels, the polymer chains are shorter, likely to result in a lower crosslinking density and, consequently, a more open and flexible network. This facilitates liquid absorption, thereby increasing their swelling capacity and favoring solvent diffusion within the hydrogel.

**Figure A1:**
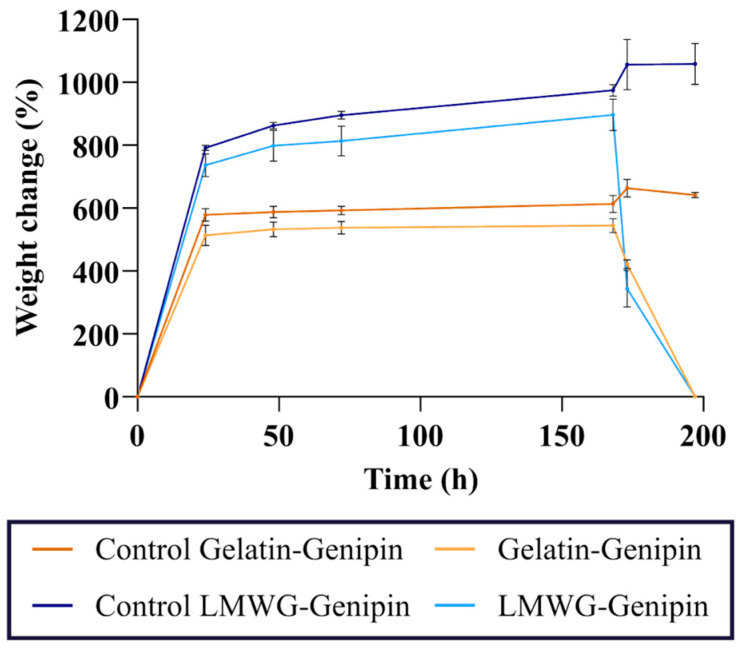
Swelling and mass loss profiles (mean ± standard deviation) for gelatin-genipin (n = 6) and LMWG-genipin hydrogels (n = 6) during enzymatic degradation with collagenase type I (10 U/mL). Data are shown together with their corresponding controls in PBS (n = 3 per hydrogel type). Both hydrogels reached complete degradation after 29 h of enzymatic exposure.

The gelatin-genipin hydrogels obtained in this work reach values similar to other gelatin hydrogels reported in the literature, which have swelling ratios between 100 and 200% [21,35]. Additionally, the swelling capacity of the hydrogels is a crucial factor for their application in drug delivery. Both gelatin-genipin and LMWG-genipin hydrogels can be considered hydrogels with high swelling capacity, as both exhibit swelling ratios greater than 150%, making them suitable for use in drug delivery. The LMWG-genipin hydrogels stand out for their superior swelling capacity, which could enable long-term drug release [36]. Also related to their potential for drug delivery applications, both gelatin-genipin and LMWG-genipin hydrogels exhibited a similar degradation behavior when exposed to collagenase type I. In both cases, a progressive loss of mass was observed, and all samples were completely degraded after 29 h of enzymatic exposure, as included in Appendix A Figure A1.

**Figure A2:**
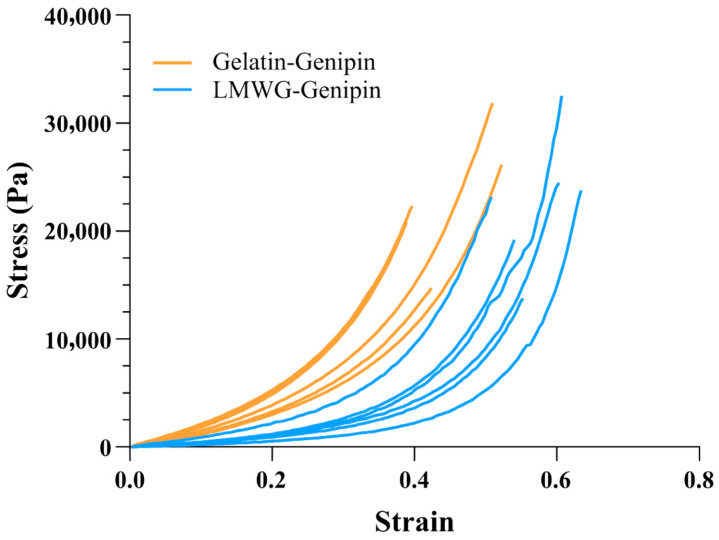
Compression test curves comparing the mechanical properties of gelatin-genipin and LMWG-genipin hydrogels (n = 6 samples for each hydrogel type).

The mechanical properties of the hydrogels were evaluated by compression tests, with the results for the maximum stress in Pa, the strain at break in %, and the elastic modulus (E) in kPa shown in Table 1. For calculating E, the linear region between 10% and 20% deformation was considered rather than the initial part of the curve. This is because in the initial phase of compression, as shown in the stress–strain curves in Appendix A Figure A2, the polymer chains can reorganize into their minimum energy configuration. Effective compression occurs when the chains align with the direction of the applied force. Significant differences were observed between the LMWG-genipin and gelatin-genipin hydrogels. Specifically, the E of the LMWG-genipin hydrogels was notably lower, measuring 6.94 ± 3.26 kPa, compared to 24.41 ± 5.55 kPa for the gelatin-genipin hydrogels, while the strain at break was higher for the LMWG-genipin hydrogels (57.41 ± 4.81) than for the gelatin-genipin hydrogels (43.07 ± 7.14). These results demonstrate the critical role of gelatin molecular weight in determining the mechanical behavior of the hydrogels. The hydrogels prepared with conventional gelatin, which contains longer polymer chains, exhibited higher crosslinking density and consequently formed stiffer and more resistant networks capable of withstanding greater stresses before failure. However, this increased rigidity was accompanied by reduced flexibility, limiting their capacity for deformation. In contrast, the LMWG-genipin hydrogels, composed of shorter chains and lower crosslinking density, generated softer and more deformable matrices with significantly higher values for the strain at break. Overall, higher molecular weight favors rigidity, whereas lower molecular weight enhances flexibility and extensibility. When compared to previously reported gelatin hydrogel formulations, the study results fall within the range of systems with lower crosslinking densities. For instance, gelatin-genipin hydrogels at 6% gelatin and 0.4% genipin had E values between 2 and 10 kPa [37], which are more consistent with the LMWG system in the present study. By contrast, markedly higher genipin concentrations, up to 5%, were reported to yield significantly enhanced properties, including strengths of ~90 kPa, deformations of 65%, and an E around 50 kPa [21]. These comparisons emphasize the strong dependence of hydrogel mechanics on both the degree of crosslinking and gelatin molecular mass. Lower crosslinker content and reduced polymer chain lengths can lead to compliant and deformable networks, while higher crosslinking densities increase stiffness and strength at the expense of extensibility. Importantly, the mechanical properties observed here are relevant in the context of mimicking the ECM. For example, the E of normal human lung parenchyma has been reported to be in the range of 0.44–7.5 kPa [38], which are values comparable to those of the LMWG-genipin hydrogels. This similarity suggests their potential suitability for applications that require compliant biomaterials capable of reproducing the mechanical environment of soft tissues.

Overall, it is important to note that the porosity, swelling ratio, and mechanical properties of the hydrogels are all interrelated. Therefore, a balance must be struck between these factors to ensure that the materials perform optimally, as the microstructure and water content strongly influence the mechanical properties of the hydrogels. For example, the gelatin-genipin hydrogels were stiffer than the LMWG-genipin hydrogels, which was consistent with the gelatin-genipin hydrogels having both smaller pore sizes and a lower swelling ratio than the LMWG-genipin hydrogels. Although LMWG has a higher free amine content, which should provide more reactive groups for crosslinking with genipin, the behavior of the LMWG-genipin hydrogels is characteristic of a lower network connectivity. One possible explanation is that the length of the polymer chains of LMWG is smaller, which limits the formation of an extensive 3D covalent network. However, longer chains, such as those of gelatin, form a more interconnected network.

### 2.4. Rheology

**Figure 5:**
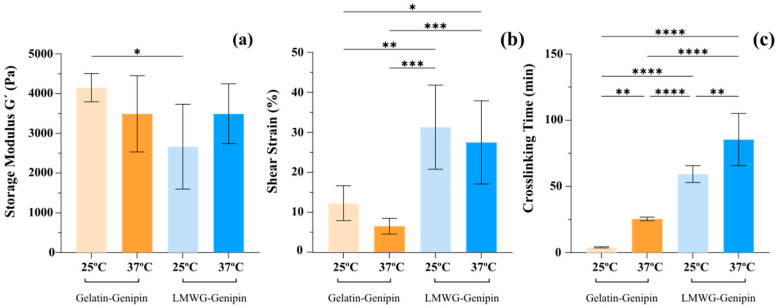
Rheological characterization of gelatin-genipin and LMWG-genipin hydrogels. (**a**) Mean values ± standard deviation (n = 6) of G’ at the LVR limit, obtained from the amplitude sweep test at 25 °C and 37 °C. (**b**) Mean values ± standard deviation (n = 6) of the shear strain in % of the LVR limit, obtained from the amplitude sweep test at 25 °C and 37 °C. (**c**) Mean values ± standard deviation (n = 6) of the gelation time at 25 °C and 37 °C. 2-way ANOVA: **** *p*-value ≤ 0.0001, *** *p*-value ≤ 0.001, ** *p*-value ≤ 0.01, and * *p*-value ≤ 0.05.

**Figure 6:**
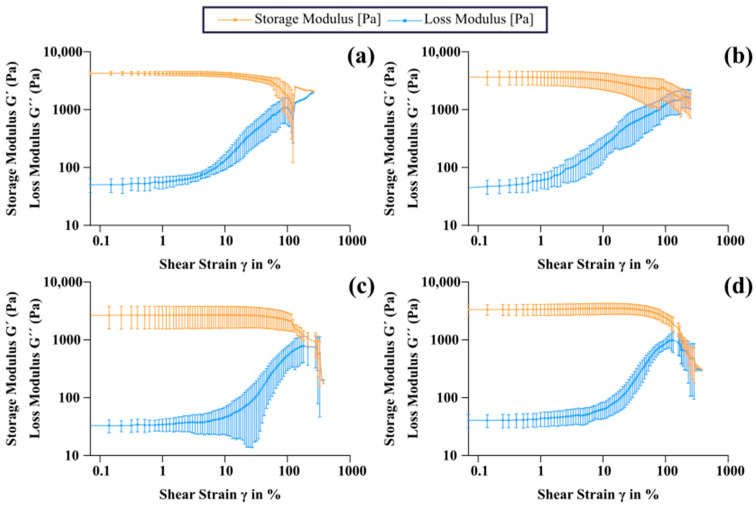
Amplitude sweep test. (**a**) Gelatin-genipin hydrogels at 25 °C. (**b**) Gelatin-genipin hydrogels at 37 °C. (**c**) LMWG-genipin hydrogels at 25 °C. (**d**) LMWG-genipin hydrogels at 37 °C. Results are shown as the mean and standard deviation of n = 6.

Rheological analysis provides a comprehensive understanding of the viscoelastic behavior of hydrogels. In this study, due to the differences in sol–gel transition temperature of gelatin and LMWG, as well as the ultimate goal of using these hydrogels in biomedical applications, the experiments were carried out both at room temperature (25 °C) and at body temperature (37 °C). First, an amplitude sweep test was carried out to understand how the hydrogel changes its mechanical response depending on the amplitude of the applied deformation, giving an overview of the structural changes in the material. In this test, the storage modulus (G’) remained constant within the linear viscoelastic region (LVR), indicating that the microstructure of the material did not undergo significant alterations under small deformations. As the applied strain increased, G’ ceased to show the initial plateau behavior and began to decrease, marking the limit of the LVR and corresponding to the beginning of the structural degradation of the material, at which point the deformation exceeds the capacity of the network to recover its original configuration. In the amplitude sweep tests performed at 25 °C, as shown in Figure 5a and Figure 6 as well as summarized in Appendix A Table A1, the G’ in the LVR was significantly higher for the gelatin-genipin hydrogels than for the LMWG-genipin hydrogels, being 4150.08 ± 356.74 Pa and 2663.38 ± 1069.71 Pa, respectively, and corroborating the compression test results at 25 °C. In contrast, the strain value for which the material ceased to have a linear behavior in the case of the LMWG-genipin hydrogels, 31.30 ± 10.53%, was significantly higher than that for the gelatin-genipin hydrogels, 12.26 ± 4.37% (Figure 5b and Appendix A Table A1), also in line with the differences in strain at break in the compression tests. In the case of experiments at 37 °C, while the deformation maintained a similar behavior with the strain value at the end of the LVR being significantly greater for the LMWG-genipin hydrogels than for the gelatin-genipin hydrogels, there were no significant differences in the G’ at 37 °C (3492.28 ± 959.86 Pa for the gelatin-genipin hydrogels and 3492.32 ± 754.63 Pa for the LMWG-genipin hydrogels). This is attributed to gelatin’s temperature sensitivity, reducing the stiffness of the material at 37 °C, whereas the LMWG-genipin hydrogels are less sensitive to temperature.

The viscoelastic behavior of the hydrogels is largely governed by the crosslinking density of the hydrogel network. Although both systems are covalently crosslinked with genipin, the extent of this covalent network depends strongly on the gelatin chain length and the availability of reactive functional groups. Supported by the results from the swelling ratio and compression measurements, the longer polymer chains in the gelatin-genipin hydrogels seem to allow the formation of a denser covalent network than the degraded gelatin in the LMWG-genipin hydrogels. This is in line with the effects of molecular weight on crosslinking that have been observed with other hydrogel systems [39,40]. In addition, gelatin contains α- and β-chains, as confirmed by the SDS-PAGE results, which are absent in LMWG. This structural feature allows the gelatin chains to interact via noncovalent interactions, further enhancing the elastic response at lower temperatures [24,40,41]. This is reflected in the rheological behavior. The gelatin-genipin hydrogels exhibit higher G′ values at 25 °C, where physical interactions are active; however, their G′ values decrease at 37 °C when these physical crosslinks are no longer effective, reaching values similar to those of the LMWG-genipin hydrogels. In contrast, LMWG-genipin hydrogels show significantly lower G′ at 25 °C because their short chains lack the ability to form physical crosslinks and do not exhibit the same thermo-sensitive behavior as conventional gelatin.

**Figure A3:**
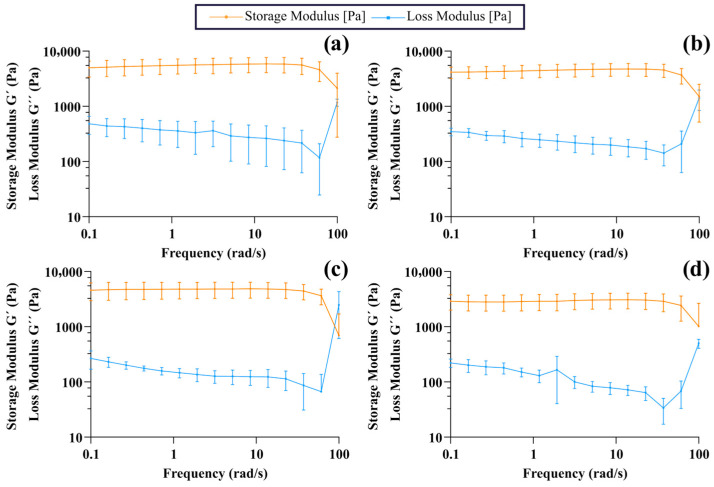
Frequency sweep test. (**a**) Gelatin-genipin hydrogels at 25 °C. (**b**) Gelatin-genipin hydrogels at 37 °C. (**c**) LMWG-genipin hydrogels at 25 °C. (**d**) LMWG-genipin hydrogels at 37 °C. Results are shown as the mean and standard deviation of n = 3.

Subsequently, a frequency sweep test was performed using a constant strain value within the previously determined LVR to select the most suitable frequency for the gelation test and to characterize the dynamic viscoelastic behavior of the materials. As shown in Appendix A Figure A3, both gelatin-genipin and LMWG-genipin hydrogels exhibited frequency-independent behavior in the range of 0.1 to 100 rad/s, with G’ and the loss modulus (G”) remaining constant. This response, typical of well-crosslinked hydrogels, indicates the presence of a stable 3D network with mechanical properties that do not depend on the deformation rate. Furthermore, this behavior was consistent at 25 °C and 37 °C for both materials, confirming that the network structure is stable across the tested temperatures.

**Figure 7:**
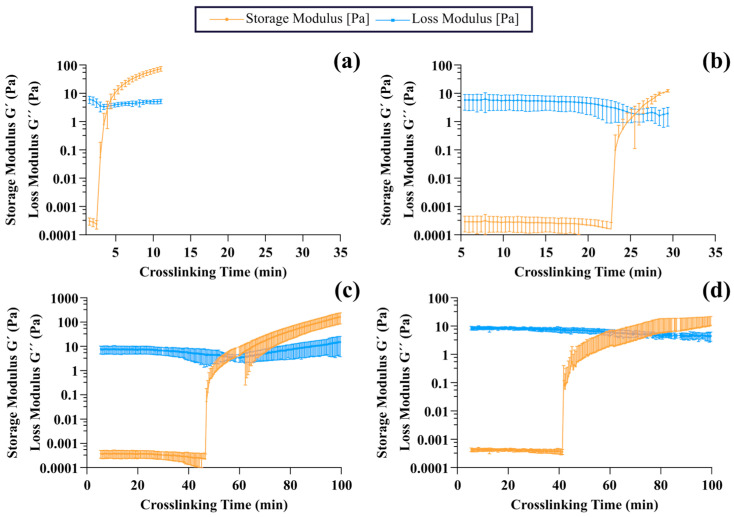
Gelation time test. (**a**) Gelatin-genipin hydrogels at 25 °C. (**b**) Gelatin-genipin hydrogels at 37 °C. (**c**) LMWG-genipin hydrogels at 25 °C. (**d**) LMWG-genipin hydrogels at 37 °C. Results are shown as the mean and standard deviation of n = 6.

Finally, the gelation times of the gelatin-genipin hydrogels and LMWG-genipin hydrogels at 25 °C and 37 °C were calculated from the crossover point between G’ and G” when the precursor solution was measured at constant values of amplitude and frequency in the LVR as determined above. As can be seen in Figure 5c and Figure 7 and Appendix A Table A2, at both 25 °C and 37 °C, crosslinking was significantly slower for the LMWG-genipin hydrogels, e.g., 59.33 ± 6.37 min at 25 °C, than for the gelatin-genipin hydrogels, e.g., 4.11 ± 0.50 min at 25 °C. In the case of the gelatin-genipin hydrogels, crosslinking at 25 °C appeared fast because the rheometer was not measuring chemical crosslinking but rather the physical crosslinking of gelatin because its sol–gel transition is above this temperature. This was confirmed by measuring the gelatin solution without adding genipin, and the crossover between G’ and G” occurred within minutes upon decreasing the temperature from 37 °C to 25 °C. However, at 37 °C, gelatin no longer exhibits this physical crosslinking, and the gelatin-genipin hydrogels took longer to gel, 25.53 ± 1.36 min, indicative of the formation of the covalent network. In the case of LMWG, this phenomenon does not occur since it is in a liquid state at both room temperature and 37 °C, and therefore, the gelation time is the time it takes for the hydrogel to form a network of covalent bonds. Comparing both materials regardless of temperature, gelation occurs earlier for gelatin, a polymer of higher molecular weight than LMWG. One potential reason is that, although LMWG has more reactive points for crosslinking with genipin due to its greater number of amino groups, the polymer chains are shorter, making it difficult to create a covalently linked network structure. This is consistent with the other characterization results with these hydrogels.

The gelation time is important in the biomedical field since the desired shape and structure of the hydrogel must be ensured. Further, if the gelation process is too fast or too slow, it can affect the viability or distribution of encapsulated cells. For example, it has been shown that with long gelation times, the cell viability decreases as the crosslinking time increases [42]. Additionally, one possible use of these hydrogels is as an ink for 3D printing or bioprinting. The crosslinking time affects the ability to print complex structures, since the layers must solidify enough to be able to hold their shape and allow the following layers to be deposited [43]. Nonetheless, the slower gelation times could be accommodated by partial gelation of the material prior to extrusion.

### 2.5. Cell Culture on the Hydrogels

**Figure 8:**
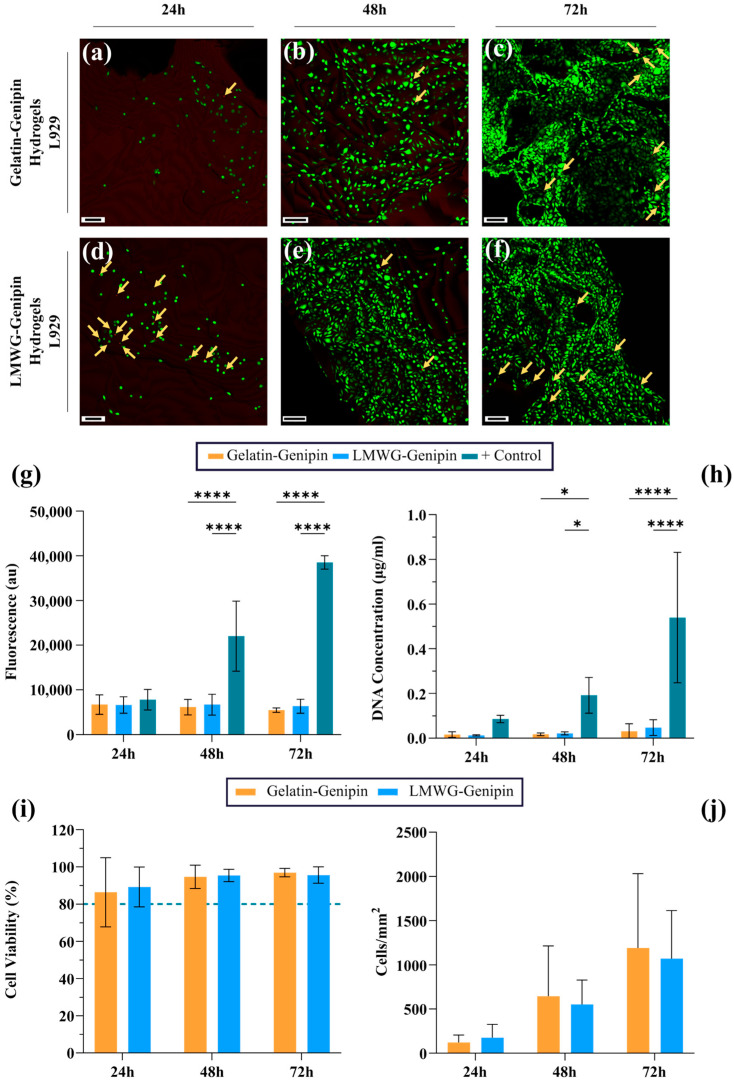
Cell viability evaluation of gelatin-genipin and LMWG-genipin hydrogels. Confocal images with live/dead staining of L929 cells, seeded at a concentration of 10^4^ cells/hydrogel. The live cells are shown in green, and in red, with yellow arrows, the dead cells. (**a**–**c**) Gelatin-genipin hydrogels, (**d**–**f**) LMWG-genipin hydrogels. Scale bars: 100 µm. (**g**) Presto Blue results. (**h**) DNA test evaluation. (**i**) Cell viability of L929 fibroblasts cultured on gelatin-genipin and LMWG-genipin hydrogels, assessed by Live/Dead staining. The green dashed line indicates the cytotoxicity threshold of 80% viability. (**j**) Quantification of cell number per area obtained from Live/Dead staining for gelatin-genipin and LMWG-genipin hydrogels. 2-way ANOVA: **** *p*-value ≤ 0.0001 and * *p*-value ≤ 0.05.

**Figure A4:**
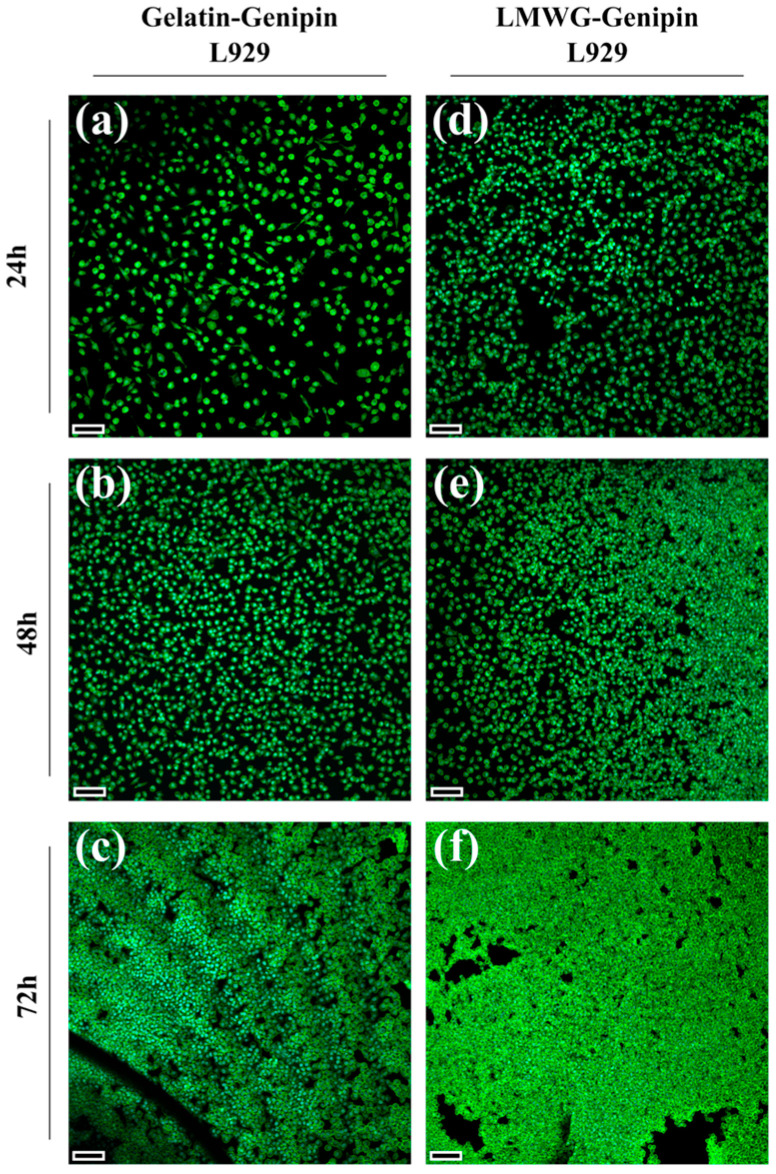
Confocal images of L929 cells for which 20,000 cells were seeded on the surface of (**a**–**c**) gelatin-genipin and (**d**–**f**) LMWG-genipin hydrogels. DAPI stains the cell nuclei (shown in blue) and Alexa Fluor 488-phalloidin stains the F-actin cytoskeleton (shown in green). Scale Bars: 100 μm.

For the biological characterization of the materials, L929 fibroblasts were used. This mouse cell line is widely used in studies of biocompatibility when testing new materials. In initial experiments, as is possible to see in Appendix A Figure A4, 20,000 cells were seeded over the hydrogel surface, and the cells reached confluence after 72 h of culture. They started changing their morphology from a spread form to a round one and exceeded a monolayer. Therefore, the number of cells seeded on each hydrogel was reduced to 10,000 to follow the growth of the cells before they reached confluence. A Live/Dead analysis was performed to both qualitatively and quantitatively evaluate the viability and proliferation of the L929 cells on the surface of the hydrogels. As shown in Figure 8a–f, for both gelatin-genipin hydrogels and LMWG-genipin hydrogels, the surface of the hydrogel was being colonized by fibroblasts from 24 h, when there was a low number of cells, until 72 h, when the surface appeared fully coated. The Live/Dead analysis was mildly affected by the autofluorescence that is produced by genipin, which has an emission wavelength over 590 nm [26] and is in the same range as the wavelength used to detect ethidium homodimer-1 (EthD-1), the fluorophore that stains dead cells. This caused a low-level background fluorescence. In addition, as is possible to see in the image, the surface of the hydrogels was not completely flat but had a certain roughness. Nonetheless, clear identification of dead cells and quantification of both live and dead cells could be performed. As shown in Figure 8i, the viability of L929 cells on both hydrogels, in general, showed high percentages of viability, indicating low cytotoxicity. For the gelatin-genipin hydrogels, viability ranged from 86.4% to 96.9%, while for the LMWG-genipin hydrogels, values between 89.3% and 95.6% were observed. Over time, no decrease in viability was detected, suggesting that both hydrogels provide an environment that is compatible with cells. Previous studies [44,45] have reported that genipin concentrations above 1% may induce cytotoxic effects. However, in the present work, a concentration of 1% (*w*/*v*) was employed to enhance the mechanical properties of the hydrogels, and an adequate level of cell viability was still observed. This suggests that the crosslinking reaction proceeded efficiently without generating toxic byproducts in amounts capable of compromising cell survival. Therefore, the results confirm that the formulation used provides a stable and biocompatible matrix, even at higher genipin concentrations, which could be advantageous for applications requiring improved mechanical performance.

Additional quantification of cell proliferation was performed using metabolic activity and DNA quantification assays. On the one hand, a study was carried out to measure the metabolic activity associated with cells adhered to the hydrogel surface, with results shown in Figure 8g. Despite the absence of significant differences at 24 h of culture, as time passed, particularly at 48 h and 72 h, a substantial difference in metabolic activity was observed between the control groups and the gelatin-genipin hydrogels and LMWG-genipin hydrogels. However, there was no significant difference between the LMWG-genipin hydrogels and the gelatin-genipin hydrogels, and there were no significant changes for these groups with time. On the other hand, a study of DNA content was conducted. As shown in Figure 8h from 24 h of culture, there was significantly less DNA concentration for the groups of gelatin-genipin hydrogels and LMWG-genipin hydrogels with respect to the control group. However, comparing both hydrogels again showed a very similar behavior between the materials. In this case, the DNA content for both hydrogel groups did show slight, but insignificant, increases with time.

Live/Dead staining qualitatively revealed cell proliferation over time, and when quantified, this showed a slight increase in cell number per area, as shown in Figure 8j. However, this trend was not statistically significant, consistent with the metabolic activity and DNA quantification results. One possible explanation for the limited proliferative response could be related to the effective number of cells attached to the hydrogel surface at the time of seeding. Due to the 3D and hydrated nature of the hydrogels, it is likely that not all cells remained on the surface after seeding, with some settling to the bottom of the well. As a result, the initial cell density in direct contact with the material may have been lower than intended, reducing cell–cell interactions and limiting proliferation. In contrast, in the positive control, all seeded cells adhered to the substrate, favoring efficient proliferation. In addition, some studies [46,47,48,49] have reported that when cells are seeded on the surface of hydrogels, proliferation during the first few days is often limited, becoming more evident after longer culture periods (around 7 days). This delayed response may be related to the time required for cells to adhere, spread, and adapt to the physicochemical and mechanical properties of the substrate.

**Figure 9:**
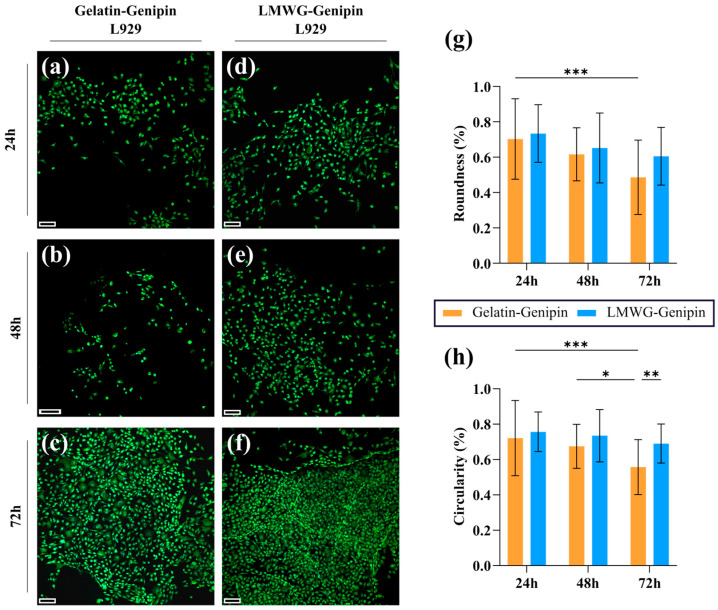
Confocal microscopy images with DAPI (shown in blue) and Alexa Fluor 488-phalloidin (shown in green) staining of L929 cells, seeded at a concentration of 10^4^ cells/hydrogel on (**a**–**c**) gelatin-genipin hydrogels and (**d**–**f**) LMWG-genipin hydrogels. Scale bars: 100 μm. (**g**) Quantification of cell morphology based on (**g**) roundness and (**h**) circularity, obtained from confocal microscopy images of DAPI/Alexa Fluor 488-phalloidin staining of L929 fibroblasts cultured on gelatin-genipin and LMWG-genipin hydrogels at 24, 48, and 72 h. 2-way ANOVA: *** *p*-value ≤ 0.001, ** *p*-value ≤ 0.01 and * *p*-value ≤ 0.05.

**Figure 10:**
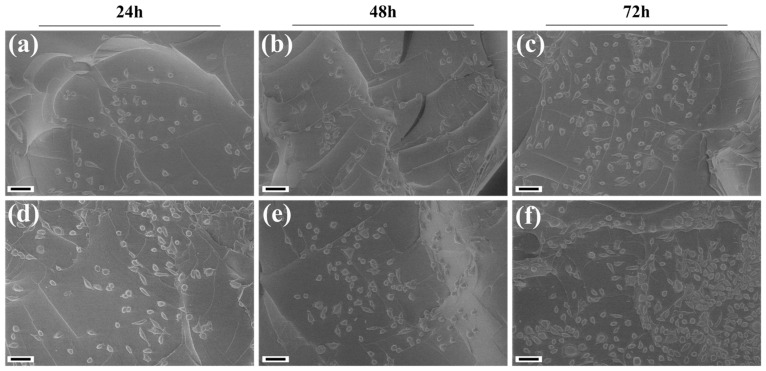
SEM images under low-vacuum conditions. (**a**–**c**) Gelatin-genipin hydrogels, (**d**–**f**) LMWG-genipin hydrogels, all with L929 cells seeded on the surface and cultured for (**a**,**d**) 24 h, (**b**,**e**) 48 h, and (**c**,**f**) 72 h. Scale bars: 50 μm.

Further evaluation was carried out through DAPI/Alexa Fluor 488-phalloidin staining (Figure 9) and SEM imaging (Figure 10). From the DAPI/Alexa Fluor 488-phalloidin images shown in Figure 9a–f, at 24 h, it was evident that the cells spread to varying degrees on the surface of the hydrogels. In some cases, the cells were extended over the surface, but in other cases, they had a rounded shape. The spreading of the cells was more visible for both hydrogels after 72 h. Additionally, SEM imaging of fixed samples (Figure 10) exhibited the same trend, in which the number of cells increased from 24h to 72 h. To confirm these observations, the roundness and circularity of the L929 cells cultured on hydrogels were calculated, as shown in Figure 9g. At 24 h, the cells showed a predominantly spherical morphology on both materials, with roundness values of 0.703 ± 0.228 and circularity values of 0.721 ± 0.212 for the cells on the gelatin-genipin hydrogels and roundness values of 0.734 ± 0.163 and circularity values of 0.757 ± 0.112 for the cells on the LMWG-genipin hydrogels, with no significant differences observed between the two groups. For the gelatin-genipin hydrogels, both roundness and circularity decreased significantly over time, reaching values of 0.486 ± 0.210 and 0.558 ± 0.155 at 72 h, indicating a progressive cell elongation. In contrast, for the LMWG-genipin hydrogels, these metrics remained higher after 72 h of culture (roundness 0.605 ± 0.163 and circularity 0.691 ± 0.110), suggesting that the cells retained a more compact morphology and spread to a lesser extent on this material. These findings indicate that matrix composition directly modulates cell morphology. While gelatin-genipin hydrogels favor a more pronounced cell elongation over time, LMWG-genipin hydrogels induce less cell extension, maintaining more rounded shapes. It is possible that the cleavage of gelatin by hydroxylamine to create the LMWG may disrupt some of the cell-adhesive peptide sequences within the gelatin chains, but this needs to be further explored.

Substrate stiffness is known to strongly influence fibroblast behavior, with optimal proliferation typically reported around 10 kPa [50]. In our study, the gelatin-genipin hydrogels exhibited a stiffness of 24.41 kPa and the LMWG-genipin hydrogels 6.94 kPa. Although these values are above and below the reported optimum, respectively, fibroblasts are capable of mechanoadaptation, gradually adjusting their morphology and cytoskeletal organization to different stiffness levels. For example, Tiskratok et al. studied L929 fibroblasts cultured on polydimethylsiloxane (PDMS) substrates with three different stiffness levels (soft, medium, and hard) [51]. Notably, the stiffness values of their soft (4.4 kPa) and hard (26 kPa) PDMS substrates are comparable to those obtained in the present study. Their work demonstrated that L929 cells exhibited a more elongated morphology on the stiffer substrate, consistent with our observations. This adaptive process may explain the absence of a significant increase at short culture times and suggests that longer incubation periods might be required to observe more pronounced cell growth on these hydrogels.

Lastly, it is important to note that the oxidative reaction that induces the color change does not affect the viability of cells grown on the surface of the hydrogels. In addition, other studies from the literature [52,53,54] confirm that there is not a problem with cell viability when cells are encapsulated within genipin-crosslinked hydrogels. However, the change in color of the hydrogel is a disadvantage, as it complicates the visualization of cells by optical microscopy and can produce background fluorescence, depending on the excitation and emission wavelengths used. It may also interfere with fluorescence-based assays, such as the PrestoBlue and PicoGreen assays used here for measuring metabolic activity and DNA content. However, further work is needed to confirm the mechanism behind this.

## 3. Conclusions

To provide a clear and concise overview of the results, Table 2 summarizes the key properties of the gelatin-genipin and LMWG-genipin hydrogels that were used throughout this work.

In this study, LMWG was successfully synthesized from conventional gelatin by hydroxylamine treatment. Hydrogels were prepared from LMWG with significantly better processability compared to those from conventional gelatin since LMWG is in a liquid state at room temperature, which facilitates its handling prior to crosslinking. Both systems achieved adequate gelation using the same concentration of genipin as a crosslinking agent. From the structural point of view, both types of hydrogels showed a porous morphology, with a slight tendency to greater porosity in the LMWG-genipin hydrogels. However, significant differences were observed in their swelling behavior. The swelling ratio was higher for the LMWG-genipin hydrogels, which is attributed to a lower density of crosslinks. This result is consistent with the mechanical properties of the hydrogels, where statistically significant differences in stiffness and deformability were identified. The gelatin-genipin hydrogels showed a higher elastic modulus and, therefore, lower deformation capacity, while the LMWG-genipin hydrogels showed a more flexible behavior. Rheologically, gelatin crosslinked with genipin had a shorter gelation time, and an additional contribution of physical solidification derived from conventional gelatin’s thermo-sensitivity was observed. Moreover, both formulations exhibited viscoelastic behavior typical of hydrogels. From the biological point of view, both types of hydrogels demonstrated good cytocompatibility in cultures with L929 cells, supporting cell proliferation over time. However, the cell proliferation observed for both hydrogels was lower than that observed for the positive control.

As both gelatin-genipin and LMWG-genipin hydrogels exhibit cytocompatibility, tunable mechanics, and suitable porosity, these features position both systems as versatile platforms for localized and prolonged therapeutic release or for supporting cell growth. Although the generated hydrogels show promising properties, there are certain limitations to consider. The crosslinking reaction with genipin, which is oxidative in nature, produces a color change in the matrix from transparent to an opaque dark blue. This transformation, although it does not affect cell viability, could hinder direct visualization of encapsulated cells by conventional optical and fluorescence microscopy techniques. Also, the time required for crosslinking may affect its potential application as a bioink in 3D printing. Despite these considerations, the results obtained position both systems as versatile and functional candidates, with potential to be optimized for tissue engineering or drug delivery applications.

## 4. Materials and Methods

### 4.1. Materials

Gelatin of porcine skin type A (bloom 300), phosphate-buffered saline (PBS) tablets, dimethyl sulfoxide (DMSO), hydroxylamine solution (50 wt% in H_2_O), sodium carbonate (Na_2_CO_3_)*,* cellulose dialysis tubing (14 kDa), sodium dodecyl sulfate (SDS)*,* sodium bicarbonate (NaHCO_3_), collagenase type I, proteinase K, and hydrochloric acid (HCl, 1 M) were obtained from Sigma Aldrich (Milwaukee, WI, USA). Genipin was from Challenge Bioproducts CO (Yun-Lin Hsien, Taiwan R.O.C.). PrestoBlue HS Cell Viability Reagent, Quant-iT™ PicoGreen™ dsDNA kit, 4′,6-diamidino-2-phenylindole (DAPI), and Alexa Fluor™ 488 Phalloidin were from Invitrogen (Waltham, MA, USA). The SureCast™ Resolving Buffer, SureCast™ Stacking Buffer, SureCast™ acrylamide (40%), SureCast™ ammonium persulfate (APS), SureCast™ tetramethylethylenediamine (TEMED), PageRuler™ Prestained Protein Ladder (from 10 to 180 kDa), Tricine SDS Sample Buffer (2X) Novex™, NuPAGE™ Sample Reducing Agent (10X)*,* PageBlue™ Protein Staining Solution, bovine serum albumin (BSA), 2,4,6-Trinitrobenzenesulfonic acid (TNBS), Dulbecco’s PBS without calcium and magnesium, TrypLE™ Express Enzyme (1X), Live/Dead Viability/Cytotoxicity Kit, paraformaldehyde solution (4% in PBS), and bovine serum albumin (BSA) were procured from Thermo Fisher Scientific (Waltham, MA, USA). Glycine and hexamethyldisilazane (HMDS) were from Tokyo Chemical Industry (TCI, Tokyo, Japan). L929 mouse fibroblasts NCTC clone 929 [L cell, L-929, derivative of Strain L] (ATCC CCL-1) were from ATCC (Manassas, VA, USA). Fetal bovine serum (FBS) was acquired from Corning (Glendale, AZ, USA). The antibiotic-antimycotic solution and Dulbecco’s Modified Eagle Medium (DMEM) were from Biowest (Nuaillé, France). Triton^®^ X-100 was from Alfa Aesar. Dulbecco’s PBS (with calcium and magnesium) was obtained from Gibco (Grand Island, NY, USA).

### 4.2. Synthesis of LMWG

First, it was necessary to prepare the LMWG through the degradation of gelatin by the action of hydroxylamine. For that, the protocol of Piluso et al. [24,25] was followed. A 100 mL aqueous solution of 1 g of gelatin was prepared and heated to 40–45 °C. Also, 100 mL of hydroxylamine solution in distilled water at 1 M was prepared. After, both solutions were mixed, and the mixture was left to incubate at 45 °C for 2 h. When necessary, the pH was adjusted to 9.4 with Na_2_CO_3_. Once finished, the solution was dialyzed against distilled water for 24 h to remove the hydroxylamine. Finally, the LMWG solution was frozen for 24 h at −80 °C and was lyophilized for 24 h to obtain the final LMWG.

### 4.3. Molecular Weight Measurements by Electrophoresis

For the determination of LMWG molecular weight compared to that of the starting gelatin, SDS-PAGE with a polyacrylamide concentration of 8%, for gelatin, and 12%, for LMWG, was performed. For the analysis, nine samples each of LMWG and conventional gelatin were dissolved in PBS at a concentration of 2.5 mg/mL. To prepare the samples for electrophoresis under denaturing conditions, 10 µL of the gelatin solution was mixed with 20 µL of Tris-Glycine SDS Sample Buffer (2X) and 4 µL of NuPAGE™ Sample Reducing Agent (10X). The volume was adjusted to 40 µL with deionized water. The samples were then heated at 85 °C for 2 min to ensure complete denaturation prior to loading onto the gel. The electrophoresis process was carried out with a current of 125 V and 40 mA for 120 min, and the PageRuler™ ladder was used to estimate the molecular weight of the proteins separated by size. After electrophoresis, the gel was carefully removed from the cassette and immediately immersed in PageBlue™ staining solution until fully covered. The gel was then incubated overnight at room temperature with gentle agitation. Following staining, the gel was rinsed with distilled water to remove excess dye.

### 4.4. Ɛ-Amino Groups Quantification by TNBS Assay

To quantify the amino groups present in the gelatin and LMWG, the TNBS assay was used following the manufacturer’s instructions. A calibration curve was prepared with known glycine concentrations from 0 to 25 μg/mL. For the test, a TNBS solution at 0.01% (*w*/*v*) in the reaction buffer, sodium bicarbonate at 0.1 M, was prepared. Then, solutions of gelatin and LMWG with concentrations of 16, 8, and 4 mg/mL were prepared, and 0.5 mL of each were mixed with 0.25 mL of the TNBS solution. This mix was incubated at 37 °C for 2 h, and the reaction was stopped by adding 0.25 mL of 10% SDS and 0.125 mL of 1 N HCl to each sample. Finally, the absorbance of the solutions was measured at 335 nm. To obtain the number of ɛ-amino groups, the concentration of the glycine standards was calculated in mol/mL, and a graph of absorbance vs. mol/mL was obtained from the calibration curve, which was used to calculate the concentration of ɛ-amino groups in the gelatin and LMWG samples.

### 4.5. Synthesis of Gelatin-Genipin Hydrogels

Hydrogels of 6 wt% protein concentration with a genipin concentration of 1 wt% were prepared. For that, 120 mg of gelatin was dissolved in 1 mL of PBS with heating to 40–45 °C, and 20 mg of genipin was dissolved in a 1:1 solution of DMSO:PBS with agitation at room temperature. After loading the mixture into a syringe, the samples were placed in an incubator at 37 °C and left to crosslink at pH 7.4 for 12–16 h (overnight).

### 4.6. Synthesis of LMWG-Genipin Hydrogels

The procedure to synthesize the LMWG-genipin hydrogels was almost the same as for the conventional gelatin-genipin hydrogels, but in this case, the dissolution of the LMWG in PBS was performed at 25 °C.

### 4.7. Swelling and Degradation Studies

Six cylindrical samples (n = 6) of 8 mm in diameter and 5 mm in thickness of each kind of hydrogel were prepared for the swelling ratio study. After 24 h of gelation in the incubator at 37 °C, the samples were individually washed in 4 mL of DMSO for 30 min to remove the genipin that did not crosslink; after that, they were washed in PBS for another 30 min to remove the residual DMSO. Finally, the samples were freeze-dried for 24 h in a SCIENTZ-12N freeze-dryer (Ningbo Scientz Biotechnology Co., Ltd., Ningbo, Zhejiang, China). After the samples were lyophilized, they were weighed and put in 2 mL of a PBS solution for the swelling study at 37 °C. The swollen hydrogels were weighed again after 3, 6, 24, 48, and 72 h in the PBS solution. The swelling ratio was calculated with the change in the hydrogel’s weight after the PBS absorption, following Equation (1):

∆S (%) = (W_S_ − W_d_)/(W_d_) × 100, (1)

where W_s_ is the weight of the hydrogel after swelling in PBS and W_d_ is the initial weight of the lyophilized hydrogel.

For degradation with collagenase type I, gelatin-genipin and LMWG-genipin hydrogels were first swollen in PBS at 37 °C until equilibrium was reached (168 h). The swollen hydrogels were then placed in 500 µL of collagenase type I solution (10 U/mL in PBS) and incubated at 37 °C. For each hydrogel type, n = 6 samples were tested with the enzyme, whereas n = 3 control samples per hydrogel type were maintained in PBS under identical conditions. Mass loss was evaluated after 5 h of incubation and again after an additional 24 h. The percentage of weight change at each time point was calculated with Equation (2):

Weight Change (%) = (W_t_ − W_d_)/(W_d_) × 100, (2)

where W_t_ is the weight of the hydrogel at the measured time point and W_d_ is the initial weight of the lyophilized hydrogel.

### 4.8. Mechanical Properties

To evaluate the mechanical properties of the hydrogels, compression tests were performed on hydrogels after 24 h of crosslinking. Six cylindrical samples (n = 6) of each type of hydrogel (gelatin-genipin and LMWG-genipin) with dimensions of 8 mm in diameter and approximately 5 mm in thickness were prepared. The preparation process involved dispensing 5 mL of hydrogel solution into 50 mm Petri dishes, which were then securely covered with parafilm to prevent drying during the overnight incubation at 37 °C. Before the compression test, the samples were extracted from the Petri dishes with an 8 mm punch and put in PBS to maintain hydration until the assay. The test was conducted on an MCR 702e Multidrive Rheometer (Anton Paar, Graz, Austria) in the Dynamic Mechanical Analysis (DMA) setup. Three samples of each hydrogel type were tested at 25 °C with a compression extensional velocity of 1 mm/min. The compression modulus was obtained with a Python (version Jupyter 6.5.2) code from the linear area of the stress–strain curves between 10 and 20% of deformation. Also, the maximum strain and stress until break were calculated.

### 4.9. Rheology

Rheological characterization was performed on the hydrogels with the Anton Paar MCR 702e Multidrive Rheometer. For the experiments, a Peltier plate was used with the 8 mm sandblasted accessory (Measuring Plate PP08/S) and a gap distance of 0.5 mm; a humidity trap was also used to avoid water evaporation from the hydrogels during the experiments. Six cylindrical hydrogels (n = 6) with a diameter of 8 mm and thickness of 0.5 mm of each formulation were tested, and the parameters used during each test are shown in Table 3. The measurements were carried out at two different temperatures, room temperature (25 °C) and body temperature (37 °C), to see how the chemical structure and the temperature affected the rheological properties. The rheological properties were evaluated with three experiments following the protocol explained by Zuidema et al. [55]. First, with an amplitude sweep test, the mean value of the end of the LVR was determined for each material in the fully formed hydrogel. In this test, the G’ and G” were evaluated until the G’ decreased. Then, a strain value inside the LVR for all hydrogels was selected to perform a frequency sweep test with a constant strain, to determine where the G’ was frequency independent. Finally, with strain and frequency values selected with the information obtained from the analysis of the two previous tests, a gelation time test was performed. In this test, the uncrosslinked solution, prepared by mixing the gelatin or LMWG solution with the genipin solution, was initially allowed to crosslink in Eppendorf tubes before being loaded into the rheometer. For most mixtures, measurements were initiated 5 min after preparation to ensure simultaneous and repeatable testing. The only exception was the gelatin-genipin mixture at 25 °C, for which measurements began 2 min after preparation due to its rapid onset of physical crosslinking resulting from its pronounced thermal sensitivity. The gelation point was identified as the time at which G’ and G” became equal, marking the onset of crosslinking.

### 4.10. Hydrogel Morphology Characterization by Scanning Electron Microscopy (SEM)

To visualize the internal morphology of the hydrogels, two samples of each type of hydrogel were prepared to obtain SEM images. First, the samples were washed with DMSO, PBS, and distilled water before freezing at −80 °C in a Laboratory Ultralow Temperature Freezer (Infrico Medcare, Lucena, Cordoba, Spain). Then, the hydrogels were lyophilized for 24 h in a FreeZone −50 °C Benchtop system (Labconco, Kansas City, MO, USA). The samples were then fractured to expose the transverse surface of the hydrogel. Afterward, they were put on aluminum pins for microscopy, glued on carbon tape, and metalized. This process was made with two cycles of 30 s at 20 mA, depositing 15 nm of gold using a Q150T sputtering machine (Quorum Technologies, Laughton, East Sussex, UK). Finally, the SEM images were taken with a FEG-SEM Apreo 2S LoVac (Thermo Fisher, Waltham, MA, USA ) at 10 kV. Pore size measurements were obtained by analyzing 50 pores per image of each hydrogel type across three SEM images.

### 4.11. Hydrogel Preparation for Cell Culture

For biological characterization, the synthesis of the hydrogels was carried out using a different procedure. The gelatin, LMWG, and genipin solutions were prepared as described earlier and filtered through polyethersulfone (PES) 0.2 mm syringe filters in a biosafety cabinet (BSC) so they were sterile. It should be noted that it was necessary to heat the gelatin solution to reduce its viscosity just before filtering. These gelatin or LMWG solutions were mixed with the genipin solutions in a Petri dish of 5 mm in diameter and allowed to gel in an incubator at 37 °C. Once gelled, the Petri dishes were washed with abundant sterile PBS, and circular hydrogels were cut with a 3D-printed punch of 8 mm diameter. The hydrogels were further washed with abundant sterile PBS for a couple of hours to remove any uncrosslinked gelatin, LMWG, or genipin. Before starting any cell culture experiment, the hydrogels were equilibrated for 30 min in complete growth medium, which was DMEM-supplemented with 10% FBS and 1% antibiotic-antimycotic solution, to prepare them for cell seeding.

### 4.12. Mouse L929 Fibroblast Culture and Seeding on Hydrogels

The L929 fibroblast cell line was maintained and expanded under standard cell culture conditions. Cells were cultured in complete growth medium. All procedures were carried out in a sterile environment using a BSC. Cells were incubated at 37 °C in a humidified atmosphere with 5% CO_2_. For subculturing, cells at approximately 80–90% confluency were washed with PBS without calcium and magnesium and detached using TrypLE™ (1X), stopping the process by adding complete growth medium. The cell suspension was then centrifuged at 100× *g* for 7 min, and the pellet was resuspended in fresh complete growth medium. Cells were counted using a hemocytometer. After cell quantification, 20,000 cells were seeded onto each hydrogel, which had been previously equilibrated in complete growth medium for 30 min. Before seeding, the medium was completely removed from the surface of the hydrogels. Cells were resuspended in 5 µL droplets and carefully deposited onto the surface of each hydrogel, as well as into the wells designated for positive controls in a 48-well plate. The plates were incubated for 30 min at 37 °C in a humidified atmosphere with 5% CO_2_ to promote cell adhesion to the hydrogel surfaces. Subsequently, 400 µL of fresh complete growth medium was gently added to each well to avoid disturbing the cells. During the characterization process, the culture medium was replaced daily to ensure optimal growth conditions and cell viability.

### 4.13. Metabolic Activity and DNA Quantification Assays

The metabolic activity of the L929 cells was assessed using the PrestoBlue assay. For this experiment, 48-well plates were prepared for each time point (24, 48, and 72 h) with three negative controls (blank), three positive controls containing the same number of cells seeded directly onto the well surface, and three test samples per formulation with cells seeded on hydrogels. At the designated time points, a solution containing 10% (*v*/*v*) PrestoBlue HS Cell Viability Reagent in complete growth medium was prepared. To eliminate interference from any cells that may have detached from the hydrogel surface and adhered to the well plate, the hydrogels were carefully transferred to a new 48-well plate before adding the reagent. Subsequently, 500 µL of the PrestoBlue solution was added to each well and incubated at 37 °C in a humidified atmosphere with 5% CO_2_ for 45 min. After incubation, 100 µL of the supernatant from each well was transferred in triplicate to a 96-well plate. The fluorescence was measured at excitation/emission wavelengths of 560 nm/590 nm using an Infinite M Plex microplate reader (Tecan, Männedorf, Switzerland).

DNA quantification was performed using the Quant-iT™ PicoGreen™ dsDNA kit, following the manufacturer’s recommendations. Prior to the analysis, the hydrogels were digested with proteinase K (0.5 mg/mL in PBS) overnight at 37 °C. After digestion, 500 µL of nuclease-free water was added, and the extracts were kept at –80 °C until processing using freeze–thaw cycles to release the DNA. The PicoGreen™ reagent was diluted 1:200 in 1X assay buffer to obtain the working solution. The standard curve was generated from a 2 μg/mL DNA stock solution, and serial dilutions were performed to obtain final concentrations of 1 μg/mL, 100 ng/mL, 10 ng/mL, and 0 ng/mL (blank). Each standard was mixed with an equivalent volume of working solution and incubated for 2–5 min at room temperature and protected from light. Once the freeze–thaw process was carried out, the samples were processed in an analogous manner, and 100 μL of each digested hydrogel was mixed with 100 μL working solution. Fluorescence was measured with a microplate reader at excitation/emission wavelengths of 480 nm/520 nm. The DNA concentration was calculated by interpolating the fluorescence values of the samples against the standard curve, after correcting for the blank values.

### 4.14. Cell Viability Quantification by Live/Dead Assay

A viability/cytotoxicity test was carried out with the Live/Dead kit. For this assay, three hydrogels per formulation were utilized for each time point (24, 48, and 72 h). At the designated time points, the medium was removed, and the hydrogels were washed three times with sterile PBS containing calcium and magnesium to maintain cell attachment to the surface. Then, a staining solution was prepared under light-protected conditions in a BSC by mixing 2 mL of PBS containing calcium and magnesium with 4 µL of ethidium homodimer-1 (EthD-1) and 1 µL of calcein-AM. Both reagents are components of the Live/Dead^®^ Viability/Cytotoxicity Kit and were used according to the manufacturer’s instructions. Subsequently, 500 µL of the staining solution was added to each hydrogel, and the samples were incubated at 37 °C with 5% CO_2_ for 30 min. Following incubation, the hydrogels were transferred to a multi-well plate with a glass bottom to facilitate imaging with an FV3000 confocal microscope (Olympus, Tokyo, Japan) with excitation/emission wavelengths of 495 nm/515 nm for calcein-AM and 495 nm/635 nm for EthD-1. After acquiring the images, the number of live and dead cells was quantified using ImageJ Fiji software (version 1.54p Java 1.8.0_452 64-bit).

### 4.15. Cell Morphology Characterization by SEM

The morphology of cells seeded on hydrogels was characterized using SEM. Cells on the hydrogel surface were fixed in 4% paraformaldehyde in PBS for 30 min and washed in PBS thrice. Then, samples were dehydrated in increasing ethanol concentrations in distilled water (30%, 50%, 70%, 90%, and 100% *v*/*v*) for 10 min each and twice in 100% ethanol. For continued dehydration, the samples were also introduced into increasing concentrations of HMDS in ethanol (33%, 50%, 66%, and 100% *v*/*v*) for 15 min each and twice in 100% HMDS. Finally, the samples were left inside a chemical hood overnight for complete drying. For imaging, the samples were mounted on the SEM pins with carbon tape, and the images were taken at a low vacuum at 5 kV.

### 4.16. Confocal Imaging of Cell Morphology

To further visualize cell adhesion and morphology, two fluorescent dyes were used. DAPI with blue fluorescence (excitation/emission wavelengths of 358 nm/461 nm) stained the cell nuclei, and Alexa Fluor 488-phalloidin with green fluorescence (excitation/emission wavelengths of 495 nm/519 nm) was used to label actin filaments in the cytoskeleton. After fixation with 4% paraformaldehyde in PBS for 30 min, the samples were washed twice with PBS and then permeabilized with 0.1% Triton X-100 in PBS for 15 min. Samples were rinsed with Dulbecco’s PBS and then incubated for 1 h with Alexa Fluor 488-phalloidin at a 1:400 dilution in PBS containing 1% BSA to reduce nonspecific background staining. Samples were washed with PBS, and cell nuclei were counterstained with 5 μg/mL DAPI for 5 min. Finally, the hydrogels were placed upside down in 24-well glass-bottom plates and viewed using a confocal microscope FV3000. Cell morphology was further characterized by quantifying circularity and roundness from the confocal images obtained after DAPI/phalloidin staining using ImageJ Fiji software. Cells cultured on the plate surface were used as a positive control. Both parameters ranged from 0 to 1. Circularity indicates whether cells maintain a regular circular outline or develop membrane protrusions. Roundness reflects how elongated the cells are. Equations (3) and (4) were used to obtain the results.

Circularity = (4 × π × Area)/(Perimeter^2^), (3)

Roundness = (4 × Area)/(π × (Major axis^2^)), (4)

### 4.17. Statistical Analysis

Statistical analysis was performed using unpaired *t*-tests and two-way analysis of variance (ANOVA), depending on the experimental design. A *p*-value ≤ 0.05 was considered statistically significant. Levels of significance are indicated as follows: **** *p*-value ≤ 0.0001, *** *p*-value ≤ 0.001, ** *p*-value ≤ 0.01, and * *p*-value ≤ 0.05.

## Figures and Tables

**Table 1:** Mechanical properties (mean ± standard deviation of n = 6 with *p*-values calculated from a *t*-test).

Hydrogel Temperature	Maximum Stress (Pa)	Strain (%)	E (kPa)
Gelatin-genipin 25 °C	21,789.67 ± 6635.42	43.07 ± 7.14	24.41 ± 5.55
LMWG-genipin 25 °C	22,813.75 ± 6210.80	57.41 ± 4.81	6.94 ± 3.26
***p*-value**	0.78817	0.00221	3.62484 × 10^−5^

**Table 2:** Overview of material characterization results for the gelatin-genipin and LMWG-genipin hydrogels developed in this study.

Properties	Gelatin-Genipin 6wt%–1wt%	LMWG-Genipin 6wt%–1wt%
Pore Diameter (μm)	31.57 ± 7.26	69.96 ± 24.19
Maximum Swelling (%)	200.30 ± 35.21	318.35 ± 17.78
Maximum Strength (kPa)	21,789.67 ± 6635.42	22,813.75 ± 6210.80
Maximum Strain (%)	43.07 ± 7.14	57.41 ± 4.81
Elastic Modulus (kPa)	24.41 ± 5.55	6.94 ± 3.26
Gelation Time (min)	25.53 ± 1.36	76.70 ± 16.81
Cell viability at 72 h (%)	96.90 ± 2.25	95.6 ± 4.41

**Table 3:** Rheology parameters used for the amplitude sweep test, the frequency sweep test, and the gelation time test for the gelatin-genipin and LMWG-genipin hydrogels.

Hydrogel	Parameter	Amplitude Sweep	Frequency Sweep	Gelation Time
Gelatin-genipin 6wt%–1wt%	T (°C)	37	37	37
	Strain (%)	1–1000	5	5
	Frequency (rad/s)	10	0.1–100	10
Gelatin-genipin 6wt%–1wt%	T (°C)	25	25	25
	Strain (%)	1–1000	5	5
	Frequency (rad/s)data	10	0.1–100	10
LMWG-genipin 6wt%–1wt%	T (°C)	37	37	37
	Strain (%)	1–1000	2	2
	Frequency (rad/s)	10	0.1–100	10
LMWG-genipin 6wt%–1wt%	T (°C)	25	25	25
	Strain (%)	1–1000	2	2
	Frequency (rad/s)data	10	0.1–100	10

## Data Availability

Data available upon reasonable request to the corresponding author.

## Abbreviations

The following abbreviations are used in this manuscript:

LMWG	Low-molecular-weight gelatin
3D	Three-dimensional
ECM	Extracellular matrix
TGF	Transforming growth factor
DMA	Dynamic mechanical analysis
ASN	Asparagine
GLY	Glycine
ξ	Mesh size
E	Elastic modulus
G’	Storage modulus
G”	Loss modulus
PBS	Phosphate-buffered saline
PDMS	Polydimethylsiloxane
DMSO	Dimethyl sulfoxide
Na_2_CO_3_	Sodium carbonate
SDS	Sodium dodecyl sulfate
NaHCO_3_	Sodium bicarbonate
HCl	Hydrochloric acid
DAPI	4,6-diamino-2-phenylindole
APS	Ammonium persulphate
TEMED	Tetramethyl ethylenediamine
BSA	Bovine serum albumin
TNBS	2,4,6-trinitrobenzanesulfonic acid
HMDS	Hexamethyldisilane
TCI	Tokyo Chemical Industry
FBS	Fetal bovine serum
DMEM	Dulbecco’s modified eagle medium
SDS-PAGE	Sodium dodecyl sulfate- polyacrylamide gel electrophoresis
SEM	Scanning electron microscopy
PES	polystyrene sulfonate
BSC	Biosafety cabinet
ANOVA	Analysis of variance

## Appendix A

**Table A1:** Amplitude sweep test of gelatin-genipin and LMWG-genipin hydrogels tested at 25 °C and 37 °C. Results are shown as the mean ± standard deviation of n = 6. Results from the statistical analysis are included, with the green and red colors indicating statistically significant and not statistically significant differences, respectively.

Hydrogel Temperature	LVR G’ (Pa)	LVR Strain (%)	Comparison	*p*-Value LVR G’	*p*-Value LVR Strain
Gelatin-genipin 25 °C	4150.08 ± 356.74	12.26 ± 4.36	Gelatin-genipin 25 °C/Gelatin-genipin 37 °C	0.5310	0.5872
LMWG-genipin 25 °C	2663.38 ± 1069.71	31.30 ± 10.53	LMWG-genipin 25 °C/LMWG-genipin 37 °C	0.3362	0.8321
Gelatin-genipin 37 °C	3492.28 ± 959.86	6.52 ± 1.97	Gelatin-genipin 25 °C/LMWG-genipin 25 °C	0.0267	0.00217
LMWG-genipin 37 °C	3492.32 ± 754.63	27.50 ± 10.42	Gelatin-genipin 37 °C/LMWG-genipin 37 °C	0.99995	0.0008

**Table A2:** Gelation times for gelatin-genipin and LMWG-genipin hydrogels tested at 25 °C and 37 °C. Results are shown as the mean ± standard deviation of n = 6. Results from the statistical analysis are included; the green color indicates a statistically significant difference.

Hydrogel Temperature	Gelation Time (min)	Comparison	*p*-Value Gelation Time
Gelatin-genipin 25 °C	4.03 ± 0.49	Gelatin-genipin 25 °C/Gelatin-genipin 37 °C	0.0092
LMWG-genipin 25 °C	59.33 ± 6.37	LMWG-genipin 25 °C/LMWG-genipin 37 °C	0.0016
Gelatin-genipin 37 °C	25.53 ± 1.36	Gelatin-genipin 25 °C/LMWG-genipin 25 °C	2.4821 × 10^−8^
LMWG-genipin 37 °C	76.70 ± 16.81	Gelatin-genipin 37 °C/LMWG-genipin 37 °C	2.28551 × 10^−5^
